# The impact of the COVID-19 pandemic on access to mental health services and socioeconomic inequalities in Italy

**DOI:** 10.3389/fpsyt.2024.1494284

**Published:** 2024-12-20

**Authors:** Alessio Petrelli, Martina Ventura, Roberta Ciampichini, Anteo Di Napoli, Valeria Fano, Christian Napoli, Martina Pacifici, Claudio Rosini, Caterina Silvestri, Fabio Voller, Alberto Zucchi, Massimiliano Aragona

**Affiliations:** ^1^ Epidemiology Unit, National Institute for Health, Migration and Poverty, Istituto Nazionale per la promozione della salute delle popolazioni Migranti e per il contrasto delle malattie della Povertà (INMP), Rome, Italy; ^2^ Epidemiology Unit, Health Protection Agency, Agenzia di Tutela della Salute (ATS), Bergamo, Italy; ^3^ Department of Public Health, Local Health Unit Roma 2, Rome, Italy; ^4^ Epidemiology Unit, Epidemiology and Cancer Registry Unit, Regina Elena National Cancer Institute, Rome, Italy; ^5^ Tuscany Regional Health Agency, Agenzia Regionale di Sanità (ARS), Florence, Italy

**Keywords:** psychiatry, mental health, mental health services, COVID-19, socioeconomic factors, immigrants

## Abstract

**Objective:**

Comprehensive evidence on the impact of the Coronavirus Disease 2019 (COVID-19) pandemic on the use of mental health services is scarce. The aim of this study was to evaluate the impact of the COVID-19 pandemic on the access to mental health services in Italy and to assess the socioeconomic and citizenship inequalities for the same outcome.

**Methods:**

A population-based longitudinal open cohort of residents aged ≥ 10 years was established in three large centers covering about 6 million beneficiaries (nearly 10% of the entire population) of the Italian National Health Service (NHS) from 01 January 2018 to 31 December 2021. The primary outcome of interest was the first access to one of the following mental health care services (FAMHS): outpatient facilities, hospital discharges, psychiatric drug prescriptions, emergency room admissions, residential and day care facilities, co-pay exemptions. To evaluate the effect of the COVID-19 pandemic on FAMHS, the temporal trend of FAMHS rates was investigated through an interrupted time series (ITS) analysis of their monthly rates. Crude incidence rates per 100,000 person days with 95%CI were calculated comparing the two time periods (pre- and post-COVID-19) by sex, age group, deprivation index (as a proxy of socioeconomic status), and citizenship. Finally, adjusted rates and rates ratios with 95%CI were estimated via ITS analysis using a step-change model.

**Results:**

ITS analysis for the trend of FAMHS rates showed a significant drop at the outbreak of the pandemic in crude rates and after adjusting for age, sex, deprivation level, and citizenship (RR=0.83 p<0.001). After the outbreak of COVID-19, the trend increased, with rates returning to pre-pandemic levels. Adjusted incidence rate ratios (IRRs) showed a higher probability of having a FAMHS for females, Italians, and for residents in the most deprived areas. A gradient of higher rates with the increase in age was observed. Greater COVID-19 impact was found on the most deprived areas of residence, with a reduction in IRRs from pre- to post-COVID-19 significantly stronger.

**Conclusions:**

The COVID-19 pandemic increased socioeconomic inequalities in mental health in Italy. Population-based cohorts are the most powerful instrument to monitor inequalities in access to mental health services and to provide timely information to drive policy.

## Introduction

The COVID-19 pandemic determined an unprecedented global crisis, creating an environment where uncertainty, life threatening conditions, and exposure to stress-inducing factors were exacerbated (1).

The pandemic hit Italy hard starting in February 2020, with different timing and intensity in the various regions of the country. An initial epidemic phase erupted in the north between February and May, then spread to the center and south especially from the fall of 2020, and continued uninterrupted, with three waves increasing intensity, until June 2021. A subsequent wave in late December 2021 was caused by the advent of the Omicron variant. The country responded by instituting several measures to contain the virus, later accompanied by the mass vaccination campaign which started on 31.12.2020.

Policy responses to the COVID-19 pandemic, particularly so-called lockdown measures (2), included the closure of non-essential activities and physical places (e.g., parks, schools), travel restrictions, and bans on social interactions, which disrupted routines and education (3). Consequent job loss and economic uncertainty further increased social insecurity. As a result, there was a growing concern about the possible negative impact of these measures on the mental health of the general population.

To mitigate the effects on the mental health of vulnerable patients, many outpatient services remained open, although their activities were reduced because national and regional rules limited interventions to the most urgent cases (4). Moreover, whenever possible, telepsychiatry was implemented for psychiatric outpatients (5). To our knowledge, specific measure to allow general practitioners to provide continuity of care to psychiatric patients were not implemented in Italy.

Excluding some local studies (6), in general, the effect of these measures is difficult to assess, and studies on the impact of the pandemic on mental health in terms of before-and-after prevalence and long-term effects have shown inconsistent results that cannot be compared due to the heterogeneity of outcomes and contexts.

A large systematic review (SR) investigated the impact of the COVID-19 pandemic on mental health in Europe by comparing it prior to and during the first phase of the pandemic; this SR showed that the prevalence of depression, generalized anxiety disorder, and non-specific mental health problems was higher during the pandemic (usually restricted to timepoints in 2020) than before the pandemic, with statistically significant increases ranging from 0.25% to 31% (7). Similar results were observed among children and adolescents (8). A high risk of bias and substantial heterogeneity among the studies led the authors of the SR to suggest caution in interpreting results (7).

Furthermore, studies evaluating the long-term impact of the pandemic found inconsistent results; a systematic review found that prevalence of anxiety, depression, PTSD, and sleep disturbances were comparable, and they did not significantly increase during the pandemic (9). On the contrary, a large English study based on 11 longitudinal cohorts found an increase in mental health disorders, although the findings were heterogeneous between cohorts and subgroups of the population (10).

Regarding the last point, the burden of the pandemic was not equally distributed in a population, with a greater deterioration in mental health in people with some vulnerabilities (11, 12) and low socioeconomic status (e.g., low education level, low income, and unemployment) (13). In addition, migrants were a group at higher risk of mental distress, given their life conditions such as displacement, poorer social support and greater isolation, problems accessing health care services, language barriers, suspension of their migration project, fear for families at home, and resident permit-related problems. Most of the studies addressing the impact of the COVID-19 pandemic on the mental health of migrant populations showed worse mental health compared to that of the native population (13, 14).

Throughout the waves of the epidemic prior to the introduction of vaccines, the massive need for assistance for COVID-19 in the most affected countries, including Italy, saturated the public and private health systems. Globally, a significant reduction in the availability and use of mental health services was observed during the first phase of the pandemic, although this varied geographically based on the spread of the pandemic in specific areas, followed by a return to normal levels (15).

In Italy, the population is covered by the National Health Service (NHS), which is tax-funded and guarantees universal access to services for all citizens. The country experienced a radical change in the organization of mental health care in 1978, with a law reform that led the transition from a hospital-based to a community-based system of care. Specifically, the health organization responsible for mental health care is the Department of Mental Health (DMH), organized into a network of community facilities that include outpatient mental health centers, general hospital psychiatric wards, day care centers, and community residential facilities. The DMH’s well-functioning information system covers the entire health care for mental disorders jointly with the other administrative health information systems.

To our knowledge, no previous study has comprehensively investigated the impact of the pandemic on the use of mental health services through a large longitudinal cohort. Accordingly, how the mental health of the Italian population changed from before to during the different waves of the COVID-19 pandemic remains poorly understood, as are the consequences for health inequalities.

The aims of this study were to evaluate the impact of the COVID-19 pandemic on the first access to mental health services (FAMHS) and to investigate whether the demographic variables, deprivation level, and citizenship affected FAMHS before and during the COVID-19 pandemic.

## Methods

This study is part of the larger COVID-19 and Mental Health (CoMeH) project (16) evaluating the impact of the COVID-19 pandemic on the use of mental health services in Italy, with a particular focus on socioeconomic and citizenship inequalities. The CoMeH project is a collaborative multicenter study promoted by the National Epidemiologic Observatory for Equity in Health (OENES) of the National Institute for Health, Migration and Poverty (INMP) and conducted in a large Italian area.

A population-based longitudinal cohort was established as an open cohort of subjects resident for at least two years, aged ≥ 10 years, and assisted by an NHS general practitioner (GP) of the area of residence in one of three large operational units: Tuscany Region (N=3,253,712), Bergamo Local Health Authorities (LHA), which covers the entire province (N=1,006,151), and the area of the Rome 2 LHA (N=907,180). The Rome 2 LHA (1,234,355 inhabitants on 31 December 2020) is the largest LHA in the Lazio region, representing 45% of the population of the Municipality of Rome. The geographic areas covered by the three participating centers account for almost 6 million beneficiaries (nearly 10% of the entire population) of the Italian National Health Service (NHS). The enrollment period was from 01 January 2018 to 31 December 2022. The data currently available are updated to 31 December 2021.

The cohort was created retrospectively from the Municipal Registries and the GP Registries of each center.

Subjects enrolled will exit the cohort at the end of the planned follow-up period (31 December 2024) or at time of death or at time of emigration, whichever occurs first. Given the open cohort design, individuals can reenter the cohort through re-immigration to the resident population of the areas of the participating centers. An extension of the follow-up period has been planned.

### Outcomes

The primary outcome of interest was the first access to one of the following mental health care services (First Access to Mental Health Services: FAMHS): outpatient facilities, hospital discharges, psychiatric drug prescriptions, emergency room admissions, residential and day care facilities, co-pay exemptions.

Specifically, one of the following six criteria had to be met for each subject aged 14 years or older to identify FAMHS: (1) at least three accesses to any mental health care service of a Department of Mental Health (DMH) within the previous 365 days; (2) at least one admission or one day care access in a residential mental health facility within the previous 365 days; (3) at least one emergency department (ED) admission or one hospitalization with a primary or secondary psychiatric diagnosis according to the International Classification of Diseases, 9^th^ revision (ICD-9); (4) being prescribed at least two psychotropic drugs from two different groups as identified in the Anatomical Therapeutic Chemical (ATC) classification system, either in the same prescription or within a maximum time frame of 30 days; (5) having received a co-pay exemption for a psychiatric disorder. It must be noted that, while the inclusion criteria (1) and (4) are not “first accesses” in the common use of the term, we preferred these criteria (more properly definable as patient intake) to increase the possibility that the measured incidence was effective and not only due to an occasional contact (e.g., a request for information or a prescription for a single psychotropic drug for non-psychiatric disorder). More details on this can be found in the Limitations section.

The complete list of the codes used is reported in **Table 1**.

**Table 1:** Criteria to identify new users of mental health services from health information systems.

Health Information Service	Enrollment criterion	Codes
**Outpatient facilities**	At least three accesses to any mental health care service of the DMHs within 365 days	–
**Hospital discharges**	At least one hospitalization with a psychiatric diagnosis	ICD9-CM codes: 291-293; 294; 294.8-294.9; 295-298; 299.90-299.91; 300-301; 303-305; 307.1; 307.40-307.49; 307.5; 307.50-307.51; 307.8; 307.80-307.81; 308; 309, 309.0; 309.1; 309.2; 309.24, 309.28, 309.29; 309.3; 309.4; 309.8; 309.81; 309.82; 309.83; 309.9; 311; 312.3; 312.30; 312.31
**Drug prescriptions**	At least two drugs for mental disorders of two different ATC groups in the same recipe or within 30 days	ATC codes: N03AE, N03AF, N03AG, N05A, N06AA, N06AB, N06AX
**Emergency department admissions**	At least one emergency department admission with a psychiatric diagnosis	ICD9-CM codes: 291.xx-293.xx; 294; 294.8-294.9; 295.xx-298.xx; 299.90-299.91; 300.xx-301.xx; 303.xx-305.xx; 307.1; 307.40-307.49; 307.5; 307.50-307.51; 307.8; 307.80-307.81; 308.xx; 309, 309.0; 309.1; 309.2; 309.24, 309.28, 309.29; 309.3; 309.4; 309.8; 309.81; 309.82; 309.83; 309.9; 311; 312.3; 312.30; 312.31
**Residential and day care facilities**	At least one hospitalization or day care access in a residential mental health facility within 365 days	–
**Co-pay exemptions**	Co-pay exemption for psychiatric disease	Exemption codes: 0.44.295-298

In order to identify “incident” accesses to mental health services, we excluded from the study those individuals who had an access according to the aforementioned criteria up to two years prior to the date of the first access to a mental health service. Accordingly, the patients included were either new cases or cases without accesses during the washout period of two years before the first registered access.

The information on accesses to mental health services was retrieved retrospectively through record linkage with the following administrative information system databases: the drug prescription database, which includes the drug prescriptions reimbursed by the NHS and dispensed from public and private pharmacies; the drug prescription registry (DP), which collects all the drugs prescribed to resident and assisted patients, both from inpatient and outpatient care settings; mental health care (MHC) databases, which include all the information regarding the interventions and activities provided by the DMH for outpatient, residential, and day care services; ED-related data; hospital discharge (HD) databases; co-pay exemptions databases.

The information from the HD database includes up to six diagnoses coded according to the ICD-9 Classification of Diseases and the date of admission and discharge. ED data include the date of access to emergency services and the principal and secondary diagnoses according to ICD-9 classification. The pharmaceutical database includes the date of prescription, the drug ATC code, and the drug quantity and unit. The MHC residential and day care facility data source includes the start and end dates of care, the type of care, diagnoses according to ICD-9 (Tuscany Region and Roma 2 LHA) or ICD-10 (Bergamo LHA), and the date and type of service.

A stepwise deterministic record linkage was performed between the demographic dataset and all health-related registries to provide combined data collected at the individual level.

Privacy protection was ensured by assigning each individual a validated anonymous patient identifier so that multiple data sources could be linked, thereby providing the study with the complete care pathway of all the citizens resident in the three geographic areas involved in the study. Any personally identifiable information was hidden from individual records. The study protocol was approved by the ethics committee of the National Institute of Health (protocol n. 0029105, 25 July 2022). Authorization for the use of anonymized data was obtained from the Data Protection Officer of all the participating centers according to EU regulation 2016/679. According to Italian law, as the study was based on administrative information system databases, individual consent was not required.

Data harmonization was carried out across centers to ensure consistent data (e.g., information was coded uniformly using the same labels and formats). The anonymized data were uploaded to an INMP server and extracted using the statistical analysis program SAS and treated in accordance with the study protocol and the instructions of the Information and Statistical System Department of the INMP.

### Covariates

Participants’ sociodemographic information, obtained from municipal population registers, included date of birth, date of death, date of emigration from or re-immigration to the residence area, municipality of residence, census tract, sex, and citizenship.

Differences by socioeconomic status were determined using the national census tract deprivation index, calculated as the sum of five standardized indicators and categorized in quintiles: low education level (% of the population with elementary school education or less); unemployment (% of the working age population that is unemployed or searching for first job); no ownership of dwelling (% of dwellings that are rented); single-parent families (% of single-parent families with minor children making up one household); residential occupant density (population per 100 square meters). A deprivation index was attributed to each subject through a record linkage with the census tract of residence. As census tracts are very small areas, the deprivation index can be considered a good proxy of individual socioeconomic status (17).

In order to investigate the impact of the pandemic on the immigrant population, the cohort was stratified based on citizenship into Italians and immigrants. All the residents in Italy without Italian citizenship were considered as immigrants. In Italy, citizenship rather than country of birth is considered the best proxy of immigrant status, at least when assessing the most recent immigrations (18); immigrants can obtain citizenship only either by marriage or by application after from three to 10 consecutive years of legal residence. Moreover, children born in Italy to foreign parents can obtain citizenship after their 18^th^ birthday.

Immigrants from highly developed countries (HDCs) were included in the *Italians* group as they accounted for only about 5% of the foreign resident population in the cohort and generally have a health profile comparable to that of the native population; immigrants from high migratory pressure countries (HMPCs) made up the *immigrants* group (19).

The period of observation was initially split into four time windows: 1) pre-pandemic, from 01 January 2018 to 21 February 2020; 2) first pandemic phase, from 22 February 2020 to 30 June 2020; 3) second pandemic phase, from 01 July 2020 to 31 December 2021. The date of 22 February 2020 corresponds to the official outbreak of the pandemic declared by the Italian authorities, while the date of 30 June 2020 corresponds to the end of the severe restrictive measures adopted in Italy. Moreover, to assess the effect of COVID-19 pandemic on FAMHS, follow-up was divided in two periods, before and after the outbreak of the COVID-19 pandemic: pre-COVID-19 (from January 2018 to February 2020) and post-COVID-19 (from March 2020).

For the purpose of the study, we considered time period, citizenship, and deprivation level as predictors; age was considered as a confounding factor and sex as an effect modifier.

### Statistical analyses

Baseline demographic and socioeconomic characteristics of the CoMeH study cohort and of the FAMHS identified during the follow-up are described as frequencies and percentages. Differences in the distribution of FAMHS based on demographic and socioeconomic characteristics, on the time period, and on the health care service of access were evaluated. The analyses were stratified by sex as the health profile of males and females, including mental health, are considerably different, as are their health care and the relative determinants.

The Chi-square test, Wilcoxon rank sum test, and Kruskal-Wallis rank sum test were used for comparisons, as appropriate. A p-value of <0.05 was considered statistically significant.

To evaluate the effect of the COVID-19 pandemic on FAMHS, the temporal trend of FAMHS rates was investigated through an ITS analysis on their monthly rates. Data were first inspected, then, according to the procedure proposed by Schuengel et al. (20), they were detrended using Loess regression and smoothing and subsequently tested for possible seasonality using a decision rule based on the Ollech-Webel test. This test classifies a time series as seasonal if either the QS‐test value is significant at p< 0.01 or the Kruskall–Wallis test is significant at p < 0.002. Any significant seasonal variation was modelled and subtracted from the raw counts to obtain residual values. Residuals were also inspected by examining their plot, the autocorrelation, and the partial autocorrelation functions and by conducting tests such as the Ljung Box test. The change in slope from the pre-COVID-19 to the post-COVID-19 period was tested using Poisson segmented regression. In order to account for residual autocorrelation and heteroscedasticity, Newey-West standard errors were calculated, and robust estimates were obtained (21).

Moreover, crude incidence rates per 100,000 person days with 95%CI were calculated comparing the two time periods (pre- and post-COVID-19) by sex, age group, deprivation level, and citizenship.

Finally, adjusted rates and rates ratios with 95%CI were estimated via an interrupted time series (ITS) analysis using a step-change model (1, 22). A negative binomial model was performed taking into account the presence of over-dispersion, with the log person time of follow-up for each group as offset. The model comprised, together with age classes and sex, a binary period term (pre-post-COVID-19) to determine the behavior step–change, citizenship, deprivation level, a linear time variable to account for trends, and a categorical calendar month variable to capture any seasonal effects. Moreover, to assess the effect of citizenship, deprivation level, and age group on FMHAS pre- and post-pandemic, three interaction terms (citizenship-time period, deprivation level-time period, and age group-time period) were included.

A sensitivity analysis was performed to further explore the interaction between age group and time period by evaluating incidence rates of FAMHS pre- and post-pandemic separately for type of health care service accessed.

All data management activities were conducted using SAS 9.3, and all statistical analyses were performed using R Studio (version 4.1.3).

## Results

Baseline characteristics of the population are shown in **Table 2**. Overall, 5,159,363 subjects were enrolled in the cohort between 1 January 2018 and 31 December 2021 from Bergamo LHA (19.5%), Rome 2 LHA (17.4%), and the Tuscany Region (63.1%). About half of the subjects were aged between 35 and 64 years, with a median age of 50 years; 52% of the population was female, and 8.7% were immigrants from HMPCs. The distribution of the population according to the quintiles of the national deprivation index showed that 23.6% of subjects lived in areas with a “middle-high” or “high” level of deprivation.

**Table 2:** Baseline characteristics of the study population.

Baseline characteristics	Population	
	N (%)	
	5,159,363	
**Age, years (median, IQR)**	50	(35, 66)
Age-groups, years		
<34	1,254,290	24.3%
35-64	2,510,617	48.7%
65-74	664,706	12.9%
75-84	506,405	9.8%
85+	223,345	4.3%
Sex		
Males	2,466,975	47.8%
Females	2,692,388	52.2%
Deprivation level (quintiles)		
Low	1,026,856	19.9%
Middle-low	1,193,167	23.1%
Middle	1,065,511	20.7%
Middle- high	753,674	14.6%
High	464,521	9.0%
Missing data	655,634	12.7%
Citizenship		
Italians	4,614,768	89.4%
Immigrants from HDCs	22,650	0.4%
Immigrants from HMPCs	446,334	8.7%
Missing data	75,611	1.5%
Participating centers		
Bergamo	1,006,151	19.5%
Rome 2	899,553	17.4%
Tuscany	3,253,659	63.1%

As shown in **Table 3**, the subjects with a FAMHS (N=206,190) had a median age of 63 years and were mainly females (61.3%). The majority of FAMHS were identified because the subject took psychotropic medications (57.2%) or had an ED admission (25.1%). The distribution by deprivation index overlapped with that of the Italian population. When considering the characteristics of FAMHS by sex, females were older and were immigrants from HMPCs in a higher percentage than were males. Moreover, the drug prescription FAMHS identifier was more frequent among females.

**Table 3:** Characteristics of incident users of mental health services, overall and by sex.

Baseline characteristics	Total		Females		Males		P-value*
	N (%)		N (%)		N (%)		
	206,190		126,456	61.3%	79,734	38.7%	
**Age, years (median, IQR)**	63	(45-80)	63	(44-79)	59	(41-77)	<0.001
Age groups, years							
14-34	32,112	15.6%	17,971	14.2%	14,141	17.7%	<0.001
35-64	73,361	35.6%	44,362	35.1%	28,999	36.4%	
65-74	27,620	13.4%	16,862	13.3%	10,758	13.5%	
75-84	39,800	19.3%	24,511	19.4%	15,289	19.2%	
85+	33,297	16.2%	22,750	18.0%	10,547	13.2%	
Citizenship							
Italians	193,178	93.7%	118,029	93.3%	75,149	94.3%	<0.001
Immigrants from HDCs	685	0.3%	486	1.1%	199	1.2%	
Immigrants from HMPCs	9,926	4.8%	6,495	5.1%	3,431	4.3%	
Missing data	2,401	1.2%	1,446	0.4%	955	0.3%	
Temporal period							
Pre-pandemic phase	122,327	59.3%	75,170	59.4%	47,157	59.1%	0.001
(from 2018-01-01 to 2020-02-21)							
First pandemic phase	14,532	7.1%	8,704	6.9%	5,828	7.3%	
(from 2020-02-22 to 2020-06-30)							
Second pandemic phase	69,331	33.6%	42,582	33.7%	26,749	33.6%	
(from 2020-07-01 to 2021-12-31)							
Health information service							
Outpatient facilities	16,665	8.1%	10,682	8.5%	5,983	7.5%	<0.001
Hospital discharges	19,014	9.2%	10,681	8.4%	8,333	10.5%	
Drugs prescriptions	117,928	57.2%	74,442	58.9%	43,486	54.5%	
Emergency room admissions	51,766	25.1%	30,225	23.9%	21,541	27.0%	
Residential and day care facilities	150	0.1%	73	0.1%	77	0.1%	
Co-pay exemptions	667	0.3%	353	0.3%	314	0.4%	
Deprivation level (quintiles)							
Low	40,771	19.8%	25,038	19.8%	15,733	19.7%	0.224
Middle-low	47,264	22.9%	28,995	22.9%	18,269	22.9%	
Middle	42,502	20.6%	26,274	20.8%	16,228	20.4%	
Middle- high	29,643	14.4%	18,122	14.3%	11,521	14.4%	
High	19,907	9.6%	12,163	9.6%	7,744	9.7%	
Missing data	26,103	12.7%	15,864	12.5%	10,239	12.8%	

*Wilcoxon rank sum test for continuous variables, Pearson's Chi-squared test for categorical variables.

**Figure 1:**
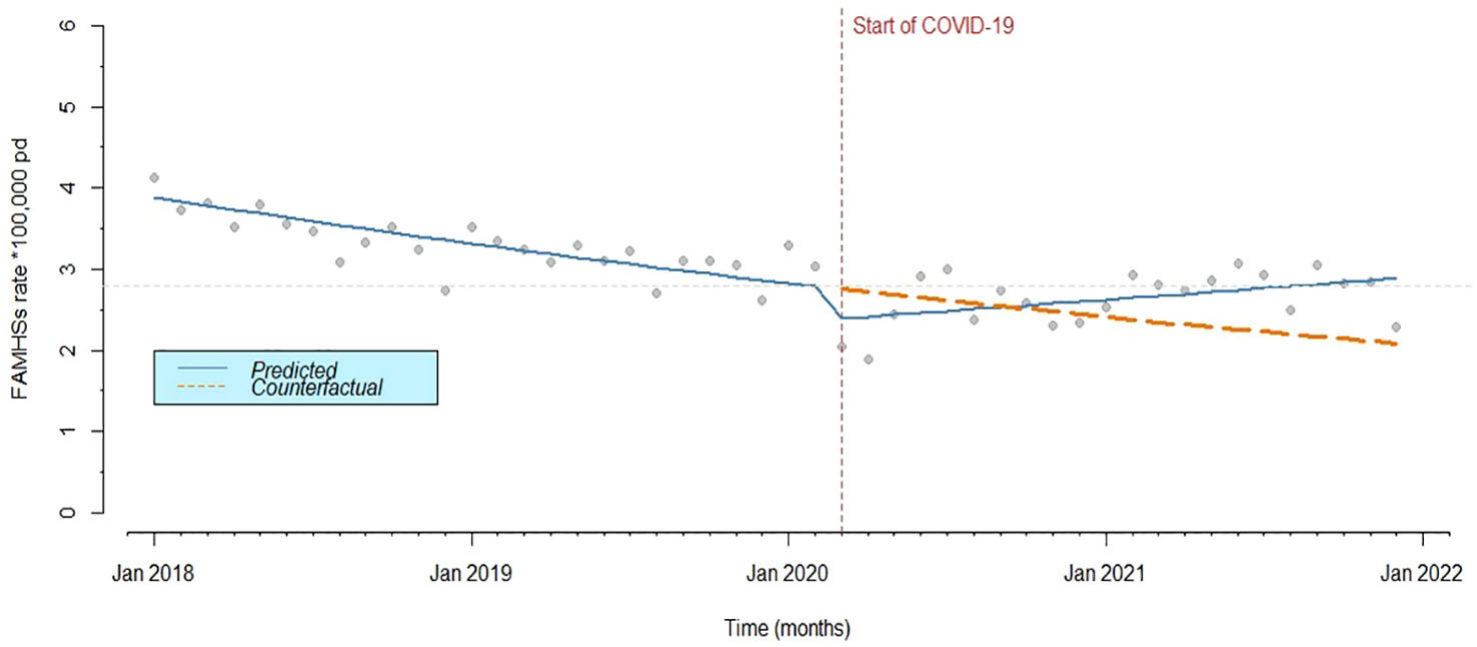
Monthly predicted rates (solid line) from ITS analysis and actual observed rates (points) of FAMHS.

**Figure 1** shows the results of the ITS analysis for the trend of FAMHS rates. In the FMHAS monthly series, no significant seasonality was detected. When examining residuals diagnostic plots, the presence of small residual autocorrelation could be hypothesized, although the Ljung-Box test was not significant (=0.09). A decreasing trend was found in the pre-COVID-19 period (IRR=0.99 [0.98-0.99]), and a significant drop was observed at the outbreak of the pandemic (IRR=0.85 [0.73-0.99]). After the outbreak of COVID-19, the trend increased (IRR=1.01 [1.00-1.02]), with rates returning to pre-pandemic levels. A decreasing trend was found in the pre-COVID-19 period, and a significant drop was observed at the outbreak of the pandemic. After the outbreak of COVID-19, the trend increased, with rates returning to pre-pandemic levels.

Crude incidence rates of FAMHS per 100,000 person days stratified by time period (pre- and post-COVID-19) and demographic and socioeconomic characteristics are reported in **Table 4**. In the pre-COVID-19 period, rates were higher in females than in males, increased with age, and were higher for those living in areas with high deprivation and in Italians compared to immigrants. Moreover, regarding the different health care services considered in the study, the highest rates of FAMHS were found for drug prescriptions and ED admissions. After the outbreak of the pandemic, an overall decrease in the FAMHS rate was observed, with a percentage reduction of 20%. This decrease involved all subgroups of mental health service users, although with different magnitudes. A higher impact of COVID-19 pandemic was found on age groups 14-34 (-22%) and 35-64 (-25%) and on immigrants from HMPCs (-26%). Furthermore, the reduction in FAMHS rates increased together with the increase in deprivation level, with the highest percentage reduction in those living in highly deprived areas (-25%). The reduction involved all health care services, with the smallest reduction being for drug prescriptions.

**Table 4:** Crude incidence rates of FAMHS per 100,000 person days by time period and demographic and socioeconomic characteristics.

	Pre COVID-19		Post COVID-19	
Sociodemographic characteristics	Users	Crude IR (95%CI) *100,000 person days	Users	Crude IR (95%CI) *100,000 person days
Total	123,322	3.29 (3.31-3.27)	82,868	2.63 (2.61-2.65)
Sex				
Male	47,551	2.66 (2.63-2.68)	32183	2.13 (2.10-2.15)
Female	75,771	3.88 (3.85-3.90)	50685	3.12 (3.07-3.12)
Age groups, years				
14-34	20,470	2.32 (2.29-2.36)	14274	1.80 (1.77-1.83)
35-64	45,785	2.46 (2.44-2.49)	29255	1.84 (1.82-1.86)
65-74	16,806	3.38 (3.33-3.43)	12589	3.09 (3.03-3.14)
75-84	23,988	6.54 (6.46-6.62)	17518	6.36 (6.26-6.45)
85+	16,273	11.46 (11.29-11.64)	9232	11.21 (10.98-11.44)
Deprivation level (quintiles)				
Low	23,863	3.19 (3.15-3.23)	16,908	2.69 (2.65-2.73)
Middle-low	28,114	3.23 (3.19-3.27)	19,150	2.62 (2.58-2.66)
Middle	25,451	3.28 (3.24-3.32)	17,051	2.62 (2.58-2.66)
Middle- high	17,901	3.27 (3.23-3.32)	11,742	2.56 (2.51-2.60)
High	12,257	3.63 (3.57-3.70)	7,650	2.73 (2.67-2.79)
Citizenship				
Italians+Immigrants from HDCs	115,853	3.40 (3.38-3.42)	78,010	2.75 (2.73-2.77)
Immigrants from HPMCs	5,890	2.04 (1.98-2.09)	4,036	1.51 (1.46-1.56)
Health care service				
Outpatient facilities	10,623	0.281 (0.275-0.286)	6,042	0.187 (0.182-0.915)
Hospital discharges	11,415	0.301 (0.296-0.307)	7,599	0.235 (0.230-0.240)
Drugs prescriptions	67,476	1.79 (1.78-1.80)	50,452	1.58 (1.57-1.59)
Emergency department admissions	33,218	0.88 (0.87-0.89)	18,548	0.58 (0.57-0.59)
Residential and day care facilities	106	0.0028 (0.0023-0.0034)	44	0.0014 (0.0009-0.0018)
Co-pay exemptions	484	0.013 (0.012-0.014)	183	0.006 (0.005-0.007)

**Figure 2:**
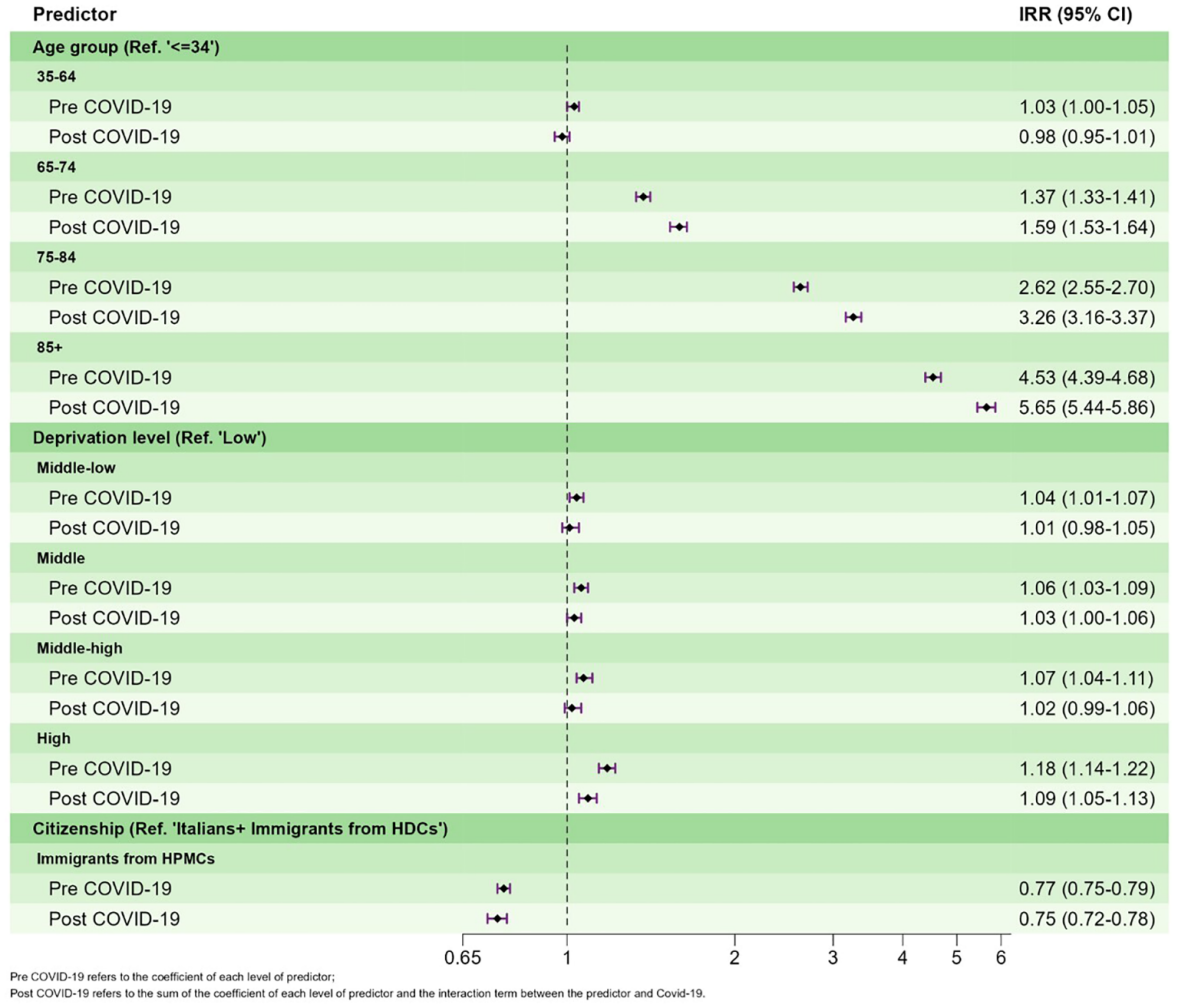
Adjusted incidence rate ratios (IRRs) of FAMHS with 95%CI pre- and post-outbreak of the COVID-19 pandemic.

The results of the multivariate ITS analysis are shown in **Table 5**. A reduction in accesses in the pandemic period compared to pre-COVID-19 was confirmed even after adjusting for age, sex, deprivation level, and citizenship (RR=0.83 p<0.001). Adjusted IRRs show that females, Italians, and residents in the most deprived areas have a higher probability of having a FAMHS. A gradient of higher rates with the increase in age was observed. Significant interactions were detected between time period and age groups and time period and deprivation level but not between time period and citizenship. A greater COVID-19 impact was found on the oldest age groups (over 75 years old) and on the most deprived areas of residence, although with opposite effects. In fact, in the period post-COVID-19, IRR of those aged 85+ (compared to the reference age class) was higher than the IRR in pre-COVID-19, whereas a reduction in IRRs from pre- to post-COVID-19 was observed for all levels of deprivation and in particular for the highest (**Figure 2**).

**Table 5:** Results of multivariate ITS analysis.

	IRR	95%CI	p-value
Time period (ref. Pre COVID-19)			
Post COVID-19	**0.83**	(0.79-0.87)	**<.0001**
**Time *(continuous)***	1.00	(0.99-1.00)	0.773
Months (ref. January)			
February	0.98	(0.95-1.01)	0.214
March	**0.92**	(0.89-0.95)	**<.0001**
April	**0.86**	(0.83-0.89)	**<.0001**
May	**0.96**	(0.93-0.99)	**0.011**
June	0.99	(0.96-1.03)	0.654
July	0.98	(0.95-1.01)	0.288
August	**0.83**	(0.80-0.86)	**<.0001**
September	**0.96**	(0.93-0.99)	**0.018**
October	**0.94**	(0.91-0.97)	**<.0001**
November	**0.91**	(0.88-0.94)	**<.0001**
December	**0.80**	(0.77-0.82)	**<.0001**
Sex (ref. Males)			
Females	**1.32**	(1.30-1.34)	**<.0001**
Age groups (ref. <=34)			
35-64	**1.03**	(1.00-1.05)	**0.037**
65-74	**1.37**	(1.33-1.41)	**<.0001**
75-84	**2.62**	(2.55-2.70)	**<.0001**
85+	**4.53**	(4.39-4.68)	**<.0001**
Citizenship (ref. Italians+HDCs)			
HMPCs	**0.77**	(0.75-0.79)	**<.0001**
Deprivation Level (ref. Low)			
Middle-low	**1.04**	(1.01-1.07)	**0.008**
Middle	**1.06**	(1.03-1.09)	**<.0001**
Middle- high	**1.07**	(1.04-1.11)	**<.0001**
High	**1.18**	(1.14-1.22)	**<.0001**
Age groups*Time period			
35-64	**0.96**	(0.92-0.99)	**0.023**
65-74	**1.16**	(1.10-1.21)	**<.0001**
75-84	**1.24**	(1.19-1.30)	**<.0001**
85+	**1.25**	(1.19-1.31)	**<.0001**
Citizenship*Time period			
HMPCs	0.97	(0.93-1.02)	0.287
Deprivation Level*Time period			
Middle-low	0.98	(0.94-1.02)	0.267
Middle	0.97	(0.93-1.01)	0.126
Middle-high	**0.95**	(0.91-1.00)	**0.031**
High	**0.92**	(0.88-0.96)	**0.001**

IRR mutually adjusted for all the variables.

Incidence rate ratios (IRRs) of FAMHS with 95%CI.

"*" stands for interaction between variables.

Bold values are those values statistically significant at p<0.05.

## Discussion

Our study showed a significant 20% decrease in the rate of FAMHS during the COVID-19 pandemic. All mental health services considered had the same trend, especially hospital admissions, emergency care, and residential and outpatient care facilities. People living in more deprived areas clearly faced stronger challenges in accessing mental health services, as the reduction was more relevant there than in the other areas. Because the rate of FAMHS had been higher in the most deprived areas before the pandemic, socioeconomic inequalities increased during the period of observation. Immigrants from HPMCs also had a strong reduction in FAHMS, greater than that in the group of Italians plus immigrants from HDCs. However, the interaction between citizenship and the time period was not significant.

The international evidence shows that the impact of the pandemic on mental health in the adult population was slight, with a moderate or null increase in the most frequent psychiatric disorders, such as anxiety, depression, PTSD, and sleep disturbances in the first few months of the pandemic and a decline in its subsequent phases (23). Nevertheless, the studies conducted on this topic were often affected by relevant bias, and the evidence regarding the long-term impact was inconsistent (9). However, a heterogeneous impact was observed among subgroups of the population (24). A reasonable hypothesis that explains these results is the barriers in access to mental health services imposed by the pandemic surveillance. In fact, the pandemic emergency caused saturation of the response capacities of the public and private health systems during the peaks of the pandemic; all non-emergency care interventions were suspended or postponed to meet the needs of COVID-19 patients. Reduced access to health care has been proven to be unfair as it penalizes the most deprived people and migrants (25), even in Italy (26, 27); this was exacerbated during the pandemic by the perception that health care facilities were potential sources of infection.

A comparison of our results with the international evidence is complicated by the geographic heterogeneity in the diffusion of the virus, the kind of restrictive measures implemented by different governments, the incidence of COVID-19 in different areas, and the characteristics of the population affected (age, socioeconomic status, being a migrant, having a previous mental disorder, and so on) (28). For example, a paper that investigated the effect of the COVID-19 pandemic on mental health visits in different countries around the world underlined the role of virtual mental health visits during the pandemic: in the countries where the shift to the telemedicine was stronger, the reduction in the health care services was lower (29). Moreover, in the first phase of the pandemic the perception of the risk of being infected in the health service settings was also higher, so that the studies conducted in 2020 showing a stronger reduction in health care services were probably more affected by these factors (28).

A Swiss study, which examined hospital discharges, outpatient services, and drug prescriptions, found a reduction in hospitalization, no impact on outpatient services, and an increase in drug use during the lockdown periods, especially among young people (30).

Most of the available studies are limited by their cross-sectional design, often conducted on small opportunistic samples, and are thus not representative of a specific population.

For instance, a reduction in the number of new patients attending mental health inpatient facilities (31–33) was observed in different countries. Also, the overall number of ED accesses decreased both immediately post-outbreak and afterward in the majority of studies (34–40), as did the number of patients attending mental health outpatient services (4, 39, 41). In Italy, the outpatient community mental health centers represent a significant part of mental health services. In the initial period of the pandemic, 25% of these centers reduced their opening hours, and 13% were temporarily closed (42). While some areas were able to maintain support for more vulnerable and severely ill patients by providing continuity of care and day-to-day support through social and supportive interventions (4), a significant drop in outpatient visits of socially disadvantaged patients, including migrants, was observed in other areas (43).

Considering the general negative impact of the pandemic, various mental health services tried to guarantee continuity of care by introducing and developing telepsychiatry, by either video call or telephone (44). As we have no data on this approach, further studies are needed to explore where and how much telepsychiatry was implemented during the pandemic and its possible effects on continuity of care.

Similarly, we need future studies exploring the possibility that online drug prescriptions mitigated the impact of the pandemic on the continuity of psychopharmacological treatments. For example, one study showed that the number of antipsychotic and mood stabilizer prescriptions in London during the pandemic remained similar to pre-pandemic numbers thanks to the extension of online prescriptions (45). However, whether this local evidence can be generalized to other sites remains to be studied.

An important result of our study regards the socioeconomic inequalities in access to mental health services. It is well known that mental health is worse in the most deprived areas (46), where the socioeconomic and/or racial composition of neighborhoods and the characteristics of the living environment appear to be correlated with an increase in mental health disorders, especially depression (47), through a complex mechanism involving the generation of more stressors for individuals residing in those areas (48). According to a recent international review of the literature, the vast majority of the evidence indicates that the pandemic has exacerbated socioeconomic inequalities in health, including mental health (13); low education level, low income, and unemployment were associated with a higher risk of psychological distress, e.g., anxiety, depression, stress symptoms, and acute stress disorders, in the vast majority of the studies included.

The widening of inequalities seems to have persisted over time. In a one-year follow-up study, the persons who had experienced economic decline were significantly less likely to report improvement in depression, anxiety, and stress (49). In a two-year study, not working and having suffered economic impact were risk factors for depressive symptoms, particularly in males (50). In November 2020, after about six months from the second COVID-19 wave, continuously precarious work or insufficient financial resources were associated with worse scores for depression, anxiety, and stress, and shifting from full-time to part-time employment was associated with higher stress and anxiety (51).

In our study, the most deprived areas experienced higher FAMHS rates pre-pandemic and the most marked reduction in FAMHS after the outbreak of the pandemic. In other words, the areas with the greatest need for health care experienced the strongest barriers to access during the pandemic, confirming an increase in health inequalities in Italy.

Regarding immigrants, first of all, it must be remembered that our classification criterion considered as Italians those immigrants from developed countries. This criterion is widely adopted in Italian studies assessing the health of immigrants, since these are people from western countries, mainly Western Europe, who have a health profile comparable to that of Italians. As this very small number of people, about 4% of the total number of immigrants, is equally distributed throughout Italy, their potential bias in epidemiological estimates is significantly limited. In our study, although we did not find any interaction between the COVID-19 pandemic and immigrant status, the FAMHS rates among immigrants were significantly lower than those of Italians, and the reduction in the access to services was higher (26% vs 19%). Accordingly, it is important to further explore possible barriers to access to health care among immigrants, especially in the outpatient setting (52).

Finally, our results show a strong direct gradient with age, with a considerable increase in the probability of using mental health services among people aged 75 or more, which may largely be explained by the age distribution of mental health distress (53). However, we cannot exclude heterogeneity by psychiatric diagnosis, which will require specific evaluation in the future. The significant interaction with the time period covariate showed that these age differences were amplified during the pandemic, highlighting a deterioration in the mental health of older individuals. As most health services were interrupted during the most critical phases of the pandemic, this result was explained by the increase in drug prescriptions, which remained easily accessible during the entire period of observation (**Supplementary Table**).

### Strengths and limitations

The main strength of our study is the design based on a large population-based cohort, followed up longitudinally through a powerful approach. This allowed us to evaluate the large amount of information regarding mental health conditions and the use of mental health services. As far as we know, this is the first study to extensively investigate the impact of the pandemic on all mental health care services by using a longitudinal approach.

A limitation of our study is the unavailability of the psychiatric diagnosis for some of the databases used in the study. While diagnosis is available for hospital discharges, ED accesses, and users of day care and residential mental health care facilities, it is not available in the drug prescription database. This could introduce a misclassification in the outcome of the study, with the risk of considering as an access to mental health care even those subjects without a mental disorder, given that psychotropic drugs are also prescribed for neurological disorders and other diseases. In any case, we believe that the criterion of having received at least two prescriptions of two different kinds of psychotropic drugs (see **Table 1**) within a maximum time frame of 30 days significantly reduced the risk of misclassification. On the other hand, the use of psychotropic drugs as an inclusion criterion for the outcome of the study was based on the deliberate choice to maximize the sensitivity of the study, as patients with mental health problems treated by GPs and private physicians would likely be included in the study if taking psychotropic medications.

The short period of the study after the pandemic did not allow us to investigate its long-term effect; however, as the cohort will be followed up until the end of 2024, this aim will be investigated in the near future.

Another limitation is the unavailability of individual information on socioeconomic status such as education level. We used an area-based indicator, the census tract deprivation index, which did not allow us to investigate the role of social factors such as living alone, lower income, or decreasing income during the pandemic, which are associated with the barriers to access of mental health care (54). Moreover, the use of an aggregate measure of socioeconomic status may have introduced residual ecological bias into the analysis.

The use of citizenship to identify immigrant status is subject to residual information bias. According to Italian legislation, individuals born in Italy to non-Italian citizens are considered foreigners until the age of 18 years, while individuals born abroad can obtain Italian citizenship if they are descendants of Italian ancestors. These two facts influence the selection of the immigrant population: while males and females born in Italy, living in Italy, and speaking Italian are included as immigrants, people born abroad who do not necessarily speak Italian, did not attend Italian schools, or have any familiarity with cultural habits and customs are included in the Italian population. Moreover, as undocumented immigrants could not be included in our sample, information on them is lacking. Furthermore, we cannot exclude a residual classification bias due to the attribution of immigrants coming from HDC in the group of Italians.

Finally, as a national institute funded to promote and coordinate projects aimed at contrasting socioeconomic inequalities, we are planning to extend the coverage of the project to a center in the south of Italy to both evaluate the impact of the pandemic on an area less affected by the pandemic and to monitor inequalities in mental health care even after the pandemic.

This study suggests practical changes that could be implemented. First of all, it highlights the importance of ensuring the continuity of mental health services even in periods of major crisis such as the recent pandemic. In particular, our study suggests that some groups are more are risk of reduced access to mental health care, so plans should be developed especially for people in deprived areas, and to some extent, for immigrants as well.

Among possible alternative plans to be developed in case of future crises is the use of telepsychiatry. Online psychopharmacological drug prescriptions must be guaranteed and extended. The enhancement of this care modality could support the outreach of certain population subgroups such as immigrants, whose difficulties in accessing services are often due to a lack of time because of work and family commitments. However, a segment of the population (e.g., the elderly, those living in socioeconomically deprived areas, and particularly marginalized subgroups such as the homeless) may have difficulty in accessing internet. In these cases, online psychopharmacological drug prescription could be enhanced by using pharmacies as proxies, where one could go to order and receive medications. Moreover, alternative forms of care should be planned, for example, home care of those patients not needing urgent hospitalization in periods when hospital beds for mental health are recruited for other uses. In addition, administering long-acting antipsychotics and home delivery of other medications can reduce the number of drop-outs and ensure continuity of care.

However, as COVID-19 will not be the last global pandemic, the primary care network plays a pivotal role as the first line of defense in any health care system. Because primary care physicians are the first point of contact with health care system for a large proportion of patients in a pandemic situation, they are involved in triage and treatment, in educating patients, and in prevention (55).

## Conclusions

The COVID-19 pandemic increased socioeconomic inequalities in access to mental health services in Italy. As the use of health services is an indirect measure of health, we can hypothesize an increase in socioeconomic inequalities in psychiatric distress as well. Regarding immigrants, our findings highlight the importance of monitoring this subgroup of population, considering their greater decrease in FAMHS.

Despite the serious economic implications of the COVID-19 pandemic, it could have been an opportunity to improve mental health care for everyone; now more than ever, it is necessary to aim at providing services that target health needs and reduce disparities. A more community-oriented MH system that guarantees low-threshold access and is more flexible in situations of crisis is desirable (56). Moreover, the risk of infection is considered lower in many community and outpatient services than in residential settings (57). Similarly, home care provides a good alternative when psychiatric hospital bed availability decrease, as they were during the pandemic because they were transformed into units to care for COVID-19 patients.

Finally, we would like to emphasize the relevance of population-based cohorts such as CoMeH to monitor inequalities in access to mental health services. These cohorts provide timely information to drive policy, both routinely and in the case of future pandemic events.

## Data Availability

The datasets presented in this article are not readily available because of data sharing legal restrictions on individual records. Requests to access the datasets should be directed to Alessio Petrelli, alessio.petrelli@inmp.it.
