# Supplementary material for: The impact of the COVID-19 pandemic on access to mental health services and socioeconomic inequalities in Italy
Source: Front Psychiatry. 2024 Dec 20;15:1494284. doi: 10.3389/fpsyt.2024.1494284 (PMC11695293; doi:10.3389/fpsyt.2024.1494284)
Supplement: Supplementary file 1 [file Table1.docx]

**Supplementary Table. Crude incidence rates of FAMHS per 100,000 person days by time period, health care service and age groups**

|  |  | **Pre COVID-19** | |  | **Post COVID-19** | |
| --- | --- | --- | --- | --- | --- | --- |
| **Health Care Service** | **Age group** | **N** | **Crude IR (95% CI) *100,000 person days** |  | **N** | **Crude IR (95% CI) *100,000 person days** |
| Outpatient facilities | 14-34 | 3044 | 0.34 (0.33-0.36) |  | 2176 | 0.27 (0.26-0.28) |
|  | 35-64 | 6078 | 0.32 (0.32-0.33) |  | 3200 | 0.20 (0.19-0.20) |
|  | 65-74 | 971 | 0.19 (0.18-0.21) |  | 478 | 0.11 (0.10-0.12) |
|  | 75-84 | 473 | 0.13 (0.11-0.14) |  | 168 | 0.06 (0.05-0.07) |
|  | 85+ | 57 | 0.04 (0.03-0.05) |  | 20 | 0.02 (0.01-0.03) |
| Hospital disharges | 14-34 | 1652 | 0.19 (0.18-0.20) |  | 1325 | 0.16 (0.16-0.17) |
|  | 35-64 | 3911 | 0.21 (0.20-0.22) |  | 2553 | 0.16 (0.15-0.16) |
|  | 65-74 | 1862 | 0.37 (0.35-0.39) |  | 1366 | 0.33 (0.31-0.34) |
|  | 75-84 | 2556 | 0.68 (0.66-0.71) |  | 1626 | 0.56 (0.53-0.59) |
|  | 85+ | 1434 | 0.98 (0.93-1.03) |  | 729 | 0.82 (0.76-0.88) |
| Drugs prescriptions | 14-34 | 5024 | 0.57 (0.55-0.58) |  | 4529 | 0.56 (0.55-0.58) |
|  | 35-64 | 21394 | 1.15 (1.13-1.16) |  | 15804 | 0.98 (0.96-1.00) |
|  | 65-74 | 10926 | 2.19 (2.14-2.23) |  | 8862 | 2.15 (2.11-2.20) |
|  | 75-84 | 17285 | 4.68 (4.61-4.75) |  | 13575 | 4.85 (4.77-4.93) |
|  | 85+ | 12847 | 8.98 (8.83-9.14) |  | 7682 | 9.16 (8.96-9.37) |
| Emergency room admissions | 14-34 | 10601 | 1.20 (1.18-1.22) |  | 6174 | 0.77 (0.75-0.79) |
|  | 35-64 | 14020 | 0.75 (0.74-0.76) |  | 7560 | 0.47 (0.46-0.48) |
|  | 65-74 | 3010 | 0.60 (0.58-0.62) |  | 1870 | 0.45 (0.43-0.47) |
|  | 75-84 | 3653 | 0.98 (0.95-1.01) |  | 2143 | 0.74 (0.71-0.77) |
|  | 85+ | 1934 | 1.32 (1.26-1.38) |  | 801 | 0.90 (0.84-0.96) |
| Residential and day care facilities | 14-34 | 31 | 0.003 (0.002-0.005) |  | 15 | 0.002 (0.001-0.003) |
|  | 35-64 | 68 | 0.004 (0.003-0.005) |  | 27 | 0.002 (0.001-0.002) |
|  | 65-74 | 4 | 0.001 (0-0.002) |  | 2 | 0 (0-0.002) |
|  | 75-84 | 3 | 0.001 (0-0.002) |  | 0 | 0 (0-0.001) |
|  | 85+ | 0 |  |  | 0 |  |
| Co-pay exemptions | 14-34 | 118 | 0.01 (0.01-0.02) |  | 55 | 0.01 (0.01-0.01) |
|  | 35-64 | 314 | 0.02 (0.01-0.02) |  | 111 | 0.01 (0.01-0.01) |
|  | 65-74 | 33 | 0.01 (0-0.01) |  | 11 | 0 (0-0) |
|  | 75-84 | 18 | 0 (0-0.01) |  | 6 | 0 (0-0) |
|  | 85+ | 1 | 0 (0-0) |  | 0 |  |
